# Sudden Death of a Pregnant Woman in Third Trimester with No Risk Factor

**DOI:** 10.1155/2012/951480

**Published:** 2012-10-22

**Authors:** Asli Goker, Melek Civi, Ozgur Bayturan, N. Kemal Kuscu

**Affiliations:** ^1^Department of Obstetrics and Gynecology, Faculty of Medicine, Celal Bayar University, 45030 Manisa, Turkey; ^2^Department of Anesthesiology, Faculty of Medicine, Celal Bayar University, 45030 Manisa, Turkey; ^3^Department of Cardiology, Faculty of Medicine, Celal Bayar University, 45030 Manisa, Turkey

## Abstract

Acute myocardial infarction in pregnancy is rare and life-threatening for both the mother and the fetus. We present the case of a 31-year-old previously healthy woman with no risk factors at 32 weeks of gestation who applied with vomiting, dyspnea and orthopnea. A respiratory arrest developed followed by loss of the fetal viability, cardiac arrest, and failure of resuscitation. We aim to raise awareness about the clinical approach to pregnant patients who are to be considered with a broad spectrum of differential diagnosis.

## 1. Introduction

 Maternal death is a sorrowful experience for the woman's family and the medical staff involved in her care. There are direct and indirect causes for maternal mortality. Preeclampsia/eclampsia, hemorrhage, sepsis, and anaphylactoid syndrome are the major causes related to pregnancy [1, 2] while cardiac disease, anesthesia complications, trauma, and cerebrovascular disease comprise indirect causes of maternal death [1, 2]. Cardiac diseases may lead to aortic dissection, myocardial infarction, sudden adult death syndrome, and peripartum cardiomyopathy resulting in approximately 10% of maternal deaths presenting as cardiac arrest [3]. 

Pregnant women applying to the emergency department tend to be evaluated by obstetricians even if their complaints are nonspecific. We present a case of sudden adult death with nonspecific symptoms and probable myocardial infarction in a previously healthy pregnant woman. We aim to point out the importance of cardiac evaluation in pregnant women when signs of suspected cardiac malfunction arise.

## 2. Case

A 31-year-old previously healthy woman, G_2 _P_1_ at 32 weeks gestation, applied to the emergency department at 4:00 PM with complaints of abdominal pain, nausea, vomiting, and shortness of breath. She reported regular visits to the obstetrician with no pregnancy-related complaints and/or complications. She had no history of systemic disease, no alcohol consumption, and no tobacco use. On arrival she was conscious, her heart rate was 85 beats per minute a blood pressure 122/78 mmHg. On physical exam, the patient had orthopnea, no edema, and a gravid abdomen. 

 On examination a viable fetus of 32 weeks, no uterine contractions, and no dilatation of the cervix were confirmed. The patient complained of back pain, neck pain, and nausea since earlier in the morning. Her first pregnancy had proceeded uncomplicated. Within minutes suddenly respiratory depression commenced. The patient became tachypneic, tachycardic, and partial oxygen pressure decreased to 65% within minutes. Respiratory arrest occurred, and the patient was intubated and hospitalized in the intensive care unit and lost consciousness. She was curarized and infused with Steradin, but deep hypotension developed and adrenalin and dopamine infusions were administered. Anuria did not respond to furosemide infusion, partial oxygen pressure became as low as 46.7%, and the fetal heart activity stopped. High doses of adrenalin and dopamine were infused. An hour later, the fetus was nonviable. 

The electrocardiogram was nonspecific. A cardiac echocardiography revealed apex and anterior left ventricle as hypokinetic and basal septum and mid septum as akinetic. A second-degree mitral insufficiency and second-degree tricuspid insufficiency were detected. There were no signs of pericardial effusion or aortic dissection. Laboratory values were as follows: CK: 877 U/L, CK-MB: 140 U/L, troponin: 40.83 ng/mL. A diagnosis of acute myocardial infarction due to segmental ventricular defect and elevated cardiac enzymes was proposed. Percutaneous transluminal coronary angioplasty (PTCA) was not performed due to acidosis, hypotension, and anuria. Over the next eight hours, the patient remained hypotensive despite vasopressor and inotropic support and electrolyte replacement and then had a cardiac arrest. After unsuccessful cardiopulmonary resuscitation, she was pronounced dead and an autopsy was declined by the family.

## 3. Discussion

Acute myocardial infarction is very rare in women of reproductive age, but the risk is increased by pregnancy. Approximately 0.4–4% of all pregnancies are complicated by cardiovascular disease, and the incidence of acute myocardial infarction is estimated as 0.6 to one in 10,000 pregnancies with the highest incidence in women over the age of 30 [4]. 

The hypercoagulable state, atherosclerosis, and coronary thrombus are reported as the most common causes of AMI during pregnancy [5, 6]. Preeclampsia also plays a contributing factor by increasing the workload of coronaries [7]. Independent risk factors for AMI in pregnancy are found as advanced age, black race, hypertension, thrombophilia, anemia, diabetes mellitus, smoking, and preeclampsia [4]. In the present case, none of these were present. 

Cases of AMI in pregnancy are reported, but usually a cause or underlying risk factor is identified such as coronary artery dissection, aortic dissection, or iatrogenic progesterone-induced vasospasm [8–10]. This case may be classified as sudden adult death syndrome (SADS) which characterises unexpected cardiac death in the community with no definite cause identified [11]. The proportion of sudden unexpected adult death is measured as 4.1% in a national survey [12]. An underlying genetic disease such as hypertrophic cardiomyopathy, dilated cardiomyopathy, and arrhythmogenic right ventricular dysplasia may lead to nonischaemic sudden cardiac death [11]. 

 Interventions such as angioplasty, stent placement or cardiopulmonary bypass are not applied frequently in pregnant women [4]. In the present case, PTCA could not be performed due to the inconvenient status of the patient. 

 Emergent cesarean delivery in the pregnant patient beyond 24 weeks of gestation is recommended in case of cardiopulmonary arrest within 4 minutes [13]. In our case, this was not possible because the fetus had lost viability long before cardiac arrest occurred. 

An autopsy would have been helpful in identifying the cause of death. In the pregnant population, acute myocardial infarction is not suspected very often. Signs and symptoms may be misinterpreted as manifestations of pregnancy. McClure concludes that symptoms of breathlessness, oedema, orthopnoea, and signs of tachypnea and tachycardia should be clues to cardiomyopathy and are indications for a chest X-ray and echocardiogram [14]. 

Maternal deaths due to vascular dissection and rupture presented with unexplainable pain, and the majority had a risk factor, namely, hypertension. Inadequate assessment of the complaints and delay in diagnosis were the most frequent problems identified in a nationwide enquiry of The Netherlands [15]. We conclude that nonspecific complaints such as back pain and shortness of breath in a pregnant woman should be evaluated profoundly. A pregnant woman applying to the hospital must be considered as a patient and not forwarded to the obstetrician immediately. This patient was fortunate to be sent back to the emergency room where respiratory arrest developed but this was not enough to save her life. 

## 4. Conclusion

Pregnant patients with nonspecific complaints must be evaluated for possible life-threatening events. This is a case report of a pregnant woman with no risk factors for myocardial infarction and shows us the importance of differential diagnosis and the unwanted consequences of delayed interventions.
